# Ferroptosis is a protective factor for the prognosis of cancer patients: a systematic review and meta-analysis

**DOI:** 10.1186/s12885-024-12369-5

**Published:** 2024-05-17

**Authors:** Shen Li, Kai Tao, Hong Yun, Jiaqing Yang, Yuanling Meng, Fan Zhang, Xuelei Ma

**Affiliations:** 1 Department of Biotherapy, West China Hospital and State Key Laboratory of Biotherapy, Sichuan University, Chengdu, Sichuan China; 2 West China School of Medicine, West China Hospital, Sichuan University, Chengdu, Sichuan China; 3 West China School of Stomatology, Sichuan University, Chengdu, Sichuan China; 4 Health Management Center, General Practice Medical Center, West China Hospital, Sichuan University, Chengdu, Sichuan China

**Keywords:** Cancer, Prognosis, Ferroptosis

## Abstract

**Background:**

Cancer is a leading global cause of death. Conventional cancer treatments like surgery, radiation, and chemotherapy have associated side effects. Ferroptosis, a nonapoptotic and iron-dependent cell death, has been identified and differs from other cell death types. Research has shown that ferroptosis can promote and inhibit tumor growth, which may have prognostic value. Given the unclear role of ferroptosis in cancer biology, this meta-analysis aims to investigate its impact on cancer prognosis.

**Methods:**

This systematic review and meta-analysis conducted searches on PubMed, Embase, and the Cochrane Library databases. Eight retrospective studies were included to compare the impact of ferroptosis inhibition and promotion on cancer patient prognosis. The primary endpoints were overall survival (OS) and progression-free survival (PFS). Studies lacking clear descriptions of hazard ratios (HR) and 95% confidence intervals for OS and PFS were excluded. Random-effects meta-analysis and meta-regression were performed on the included study data to assess prognosis differences between the experimental and control groups. Meta-analysis results included HR and 95% confidence intervals.

This study has been registered with PROSPERO, CRD 42023463720 on September 27, 2023.

**Results:**

A total of 2,446 articles were screened, resulting in the inclusion of 5 articles with 938 eligible subjects. Eight studies were included in the meta-analysis after bias exclusion. The meta-analysis, after bias exclusion, demonstrated that promoting ferroptosis could increase cancer patients’ overall survival (HR 0.31, 95% CI 0.21–0.44) and progression-free survival (HR 0.26, 95% CI 0.16–0.44) compared to ferroptosis inhibition. The results showed moderate heterogeneity, suggesting that biological activities promoting cancer cell ferroptosis are beneficial for cancer patient’s prognosis.

**Conclusions:**

This systematic review and meta-analysis demonstrated that the promotion of ferroptosis yields substantial benefits for cancer prognosis. These findings underscore the untapped potential of ferroptosis as an innovative anti-tumor therapeutic strategy, capable of addressing challenges related to drug resistance, limited therapeutic efficacy, and unfavorable prognosis in cancer treatment.

**Registration:**

CRD42023463720.

**Supplementary Information:**

The online version contains supplementary material available at 10.1186/s12885-024-12369-5.

## Background

Cancer has progressively become the world’s leading cause of mortality, imposing substantial disease burdens. According to GLOBOCAN 2020, the global cancer burden will reach 28.4 million cases in 2040 [1]. And approximately one in every five men and one in every six women will develop cancer, with one in eight men and one in ten women succumbing to cancer before reaching 75 years of age [2]. It is estimated that over half of all cancer-related deaths (57.3%) and nearly half of all new cancer cases (48.4%) are concentrated in Asia [2]. Presently, common treatments for cancer encompass surgery, radiation, and chemotherapy [3, 4]. However, these approaches may harm normal cells and result in significant side effects, including hepatotoxicity, ototoxicity, cardiotoxicity, nausea, vomiting, and more [5, 6]. Despite advancements in therapy, cancer remains the second leading global cause of death, following ischemic heart disease, and is projected to become the leading cause by 2060 [7].

In 2012, a nonapoptotic, iron-dependent form of cell death initiated by the oncogenic Ras-selective lethal small molecule erastin was termed “ferroptosis” [8]. Ferroptosis exhibits distinct morphological characteristics compared to other regulated cell death forms. Notably, ferroptosis lacks the hallmark signs of apoptosis, such as chromatin condensation and apoptotic bodies, instead manifesting as shrunken mitochondria, reduced mitochondrial cristae, and an accumulation of lipid peroxides [8–10]. Its underlying mechanism also differs from other regulated cell death processes. Ferroptosis is inhibited by the system xc-—GSH—GPX4 pathway and is induced by the accumulation of phospholipid hydroperoxides, rather than the involvement of cell death executioner proteins such as caspases and mixed lineage kinase domain-like protein, among others [9, 11].

An increasing body of research has explored the role of ferroptosis in tumors, suggesting its dual role in tumor promotion and inhibition. Various experimental agents, including erastin, RSL3, and drugs such as sorafenib, sulfasalazine, statins, and artemisinin, along with ionizing radiation and cytokines like IFN-γ and TGF-β1, can induce ferroptosis and inhibit tumors [12]. However, emerging evidence hints at ferroptosis potentially promoting tumor growth by triggering inflammation-associated immunosuppression within the tumor microenvironment [12, 13]. Numerous studies have also indicated the prognostic value of ferroptosis [14–18].

Given the unclear role of ferroptosis in cancer biology, we conducted this meta-analysis to investigate its impact on cancer prognosis.

## Method

### Search strategy and selection criteria

This systematic review and meta-analysis were conducted following PRISMA guidelines. PubMed, EMBASE, and the Cochrane Library were systematically searched from their inception until February 27, 2024, with no language restrictions. The search strategy included the following terms: (ferroptosis or oxytosis) AND (Neoplasm or Tumor or Tumors or Neoplasia or Cancer or Cancers or Malignant Neoplasm or Malignancy or Malignant Neoplasms or Neoplasms, Malignant or Benign Neoplasms or Neoplasm, Benign or Malignancies or Neoplasm, Malignant or Benign Neoplasm or Neoplasms, Benign or Neoplasias) AND (prognosis or Prognoses or Prognostic Factors or Prognostic Factor or Factor, Prognostic or Factors, Prognostic) as free text.

The objective of this study is to investigate and elucidate the impact of ferroptosis on cancer patients’ prognosis. We will compare the differences in prognosis between cancer patients with genes that promote ferroptosis and those with genes that inhibit it. The primary endpoints of the study include HRs and 95% confidence intervals for OS and PFS. It is important to note that the upregulation and downregulation of ferroptosis-related genes are not used as criteria for grouping; rather, the experimental and control groups are divided based on the ultimate impact of genes on ferroptosis. This meta-analysis was limited to studies conducted in humans. Participant data from cohort studies were extracted and analyzed. The collected information included the first author, study period, country of study, study size, ferroptosis-related gene, the effect of genes on ferroptosis, type of cancer, HR, and 95% confidence intervals for OS and PFS.

Both exclusion and inclusion criteria were pre-specified. Studies demonstrating a relationship between prognosis and ferroptosis in cancer patients were selected. Inclusion criteria were as follows: (1) Articles were limited to those involving human samples only. (2) All cancer patients had been diagnosed by pathological evidence. (3) Expression of ferroptosis-related genes had been assessed through immunohistochemistry from tumor specimens, conducted according to standard protocols. (4) All patients had been subject to follow-up, and results had been reported. Exclusion criteria encompassed: (1) Duplicate articles. (2) Article types other than original research, such as reviews, meta-analyses, letters, or editorial comments. (3) Studies involving cellular or animal-based research. (4) Patients with multiple primary cancers. The literature search, study selection, and data extraction were independently performed by Shen Li and Kai Tao, with any discrepancies reviewed and resolved by another author, Xuelei Ma, through consensus.

### Data analysis

We employed Stata 14 software to calculate statistics. The specific analysis method is as follows: (1) We collected and analyzed the HR for OS and PFS reported in the included studies. The results were visualized using forest plots to illustrate the differences in prognosis between cancer patients whose genes promote ferroptosis and those whose genes inhibit it, thereby demonstrating the impact of ferroptosis on the prognosis of cancer patients. (2) Heterogeneity test was conducted by I^2^ statistic to assess the heterogeneity of the results. Low heterogeneity was defined as an I^2^ value less than or equal to 25%, moderate heterogeneity as between 25 and 75%, and high heterogeneity as exceeding 75%. (3) To evaluate potential publication bias, we employed funnel plots and conducted Egger tests. A *p*-value greater than 0.05 in Egger test indicates no significant bias. (4) Sensitivity analysis was conducted to examine any studies with significant influence on the overall results. (5) Meta-regression was conducted to assess the potential influence of covariates on the outcome [19, 20]. We subjected the included covariates to regression testing, including country of study, ferroptosis-related gene, the effect of genes on ferroptosis, and type of cancer, to explore possible sources of heterogeneity and reduce potential bias. This study has been registered with PROSPERO, CRD 42023463720.

### Bias analysis and quality assessment

Three researchers (LS, YJQ and TK) independently conducted a bias risk assessment following the Cochrane Bias Assessment Handbook. Considering that all included studies were retrospective articles, this study employed the Cochrane bias risk tool, which comprises five domains, to evaluate the risk of bias in each included study: (1) selection bias, (2) measurement bias, (3) data integrity bias, (4) outcome selection bias, and (5) other biases. Each researcher independently assessed the risk as low, high, or unclear for each domain. In cases of any uncertainty, Dr. Xuelei Ma made the final judgment. Based on the risk of bias, the quality of evidence was categorized as very low, low, moderate, or high. The quality assessment of this study adheres to the GRADE system.

## Results

**Fig. 1:**
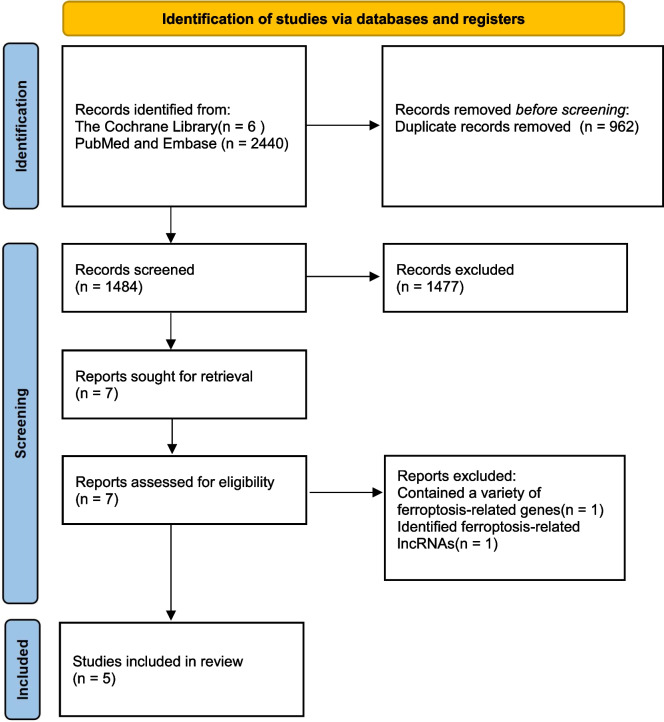
Study selection

We identified a total of 2,446 articles through literature searches, with 6 articles from the Cochrane Library and 2,440 from other databases, including PubMed and Embase. We excluded 962 duplicate articles. Among the remaining literature, we excluded 1,477 articles after abstract screening as they did not align with our research objectives. Subsequently, we conducted full-text reviews and eligibility assessments on the remaining 7 articles. Ultimately, we included 5 articles in our analysis. The review process was conducted independently by LS, TK and MYL, with a third reviewer, Xuelei Ma, reassessing articles with uncertain eligibility. The process is illustrated in Fig. 1.

Among the five clinical articles, all studies were conducted in Asia, with 2 studies in China (40%) and 3 in Japan (60%). The research covered various cancer types, including gastric cancer and esophageal cancer of the digestive system, epithelial ovarian cancer of the female reproductive system, and osteosarcoma originating from undifferentiated bone fibrous tissue. In terms of age reporting, the median age of patients with epithelial ovarian cancer was 52 years, while osteosarcoma patients had an average age of 30.2 years, which is consistent with the characteristics of these two diseases. Three out of the five articles included two studies each, resulting in a total of 8 studies. Glutathione peroxidase 4 (GPX4) was the most studied gene (4/8, 50%) related to regulating ferroptosis. Like most other genes, GPX4 plays a role in inhibiting ferroptosis by suppressing lipid peroxidation. In contrast, heme oxygenase 1 (HMOX1), through catalyzing the degradation of heme into divalent iron ions, biliverdin, and CO, can promote ferroptosis by increasing the labile iron pool (LIP) (1/8, 12.5%). It’s worth noting that, as shown in Table 1, only 3 studies (3/8, 37.5%) reported cut-off values, while the rest did not report them. We will discuss the importance of this missing data in the Discussion section.

**Table 1:** Study characteristics for included studies

**Study (Year)**	**Study period**	**Country**	Number	Age(mean ± SD or median(range))	**Number(high risk: low risk)**	**Ferroptosis related gene**	The effect of genes on ferroptosis	**Type of cancer**	Cut off	**HR (95% CI) for OS**	**HR (95% CI) for PFS**
Shishido (2021) [21]	2009–2017	Japan	97	NA	40: 57	GPX4	negative	ESCC	50%	0.1(0.05,0.21)	0.09(0.04,0.19)
Shishido (2021) [21]	2009–2017	Japan	97	NA	51: 45	HMOX1	positive	ESCC	50%	0.217(0.106,0.445)	0.21(0.104,0.426)
Song (2021) [22]	2011–2016	China	60	30.2 ± 5.8	27: 33	ZFP36	negative	osteosarcoma	NA	7.65(3.02,19.4)	12.86(5.16,32.02)
Sugezawa (2022) [23]	2004–2011	Japan	106	NA	63: 43	GPX4	negative	gastric cancer	25%	0.449(0.235,0.858)	0.336(0.136,0.828)
Wu (2022) [24]	2002–2018	China	192	52(31–72)	43: 149	SLC7A11	negative	epithelial ovarian cancer	NA	0.35(0.202,0.606)	0.427(0.272,0.671)
Wu (2022) [24]	2002–2018	China	192	52(31–72)	44: 148	GPX4	negative	epithelial ovarian cancer	NA	0.445(0.257,0.77)	0.364(0.228,0.583)
Miyauchi (2023) [25]	2009–2017	Japan	97	NA	20: 77	FSP1	negative	ESCC	NA	0.419(0.207,0.848)	NA
Miyauchi (2023) [25]	2009–2017	Japan	97	NA	40: 57	GPX4	negative	ESCC	NA	0.326(0.185,0.576)	NA

*GPX4* Glutathione peroxidase 4, *HMOX1* Heme oxygenase 1, *ZFP36* Zinc finger protein 36, *FSP1* Ferroptosis suppressor protein 1, *ESCC* Esophageal squamous cell carcinoma

### Main outcome

**Fig. 2:**
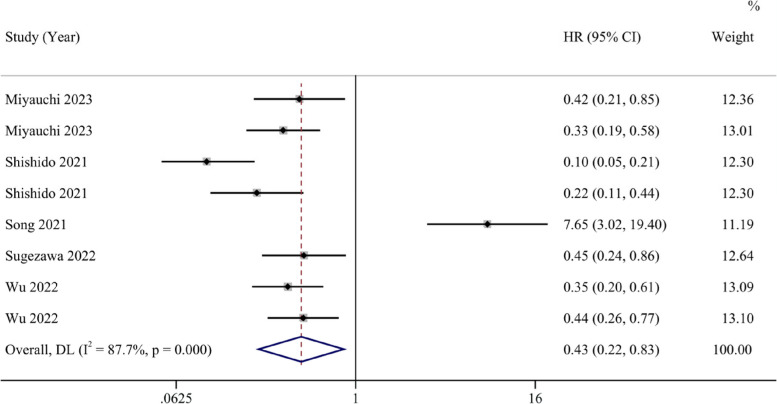
Forest plot of the pooled overall survival between the ferroptosis-promoting group and the ferroptosis-inhibiting group

**Fig. 3:**
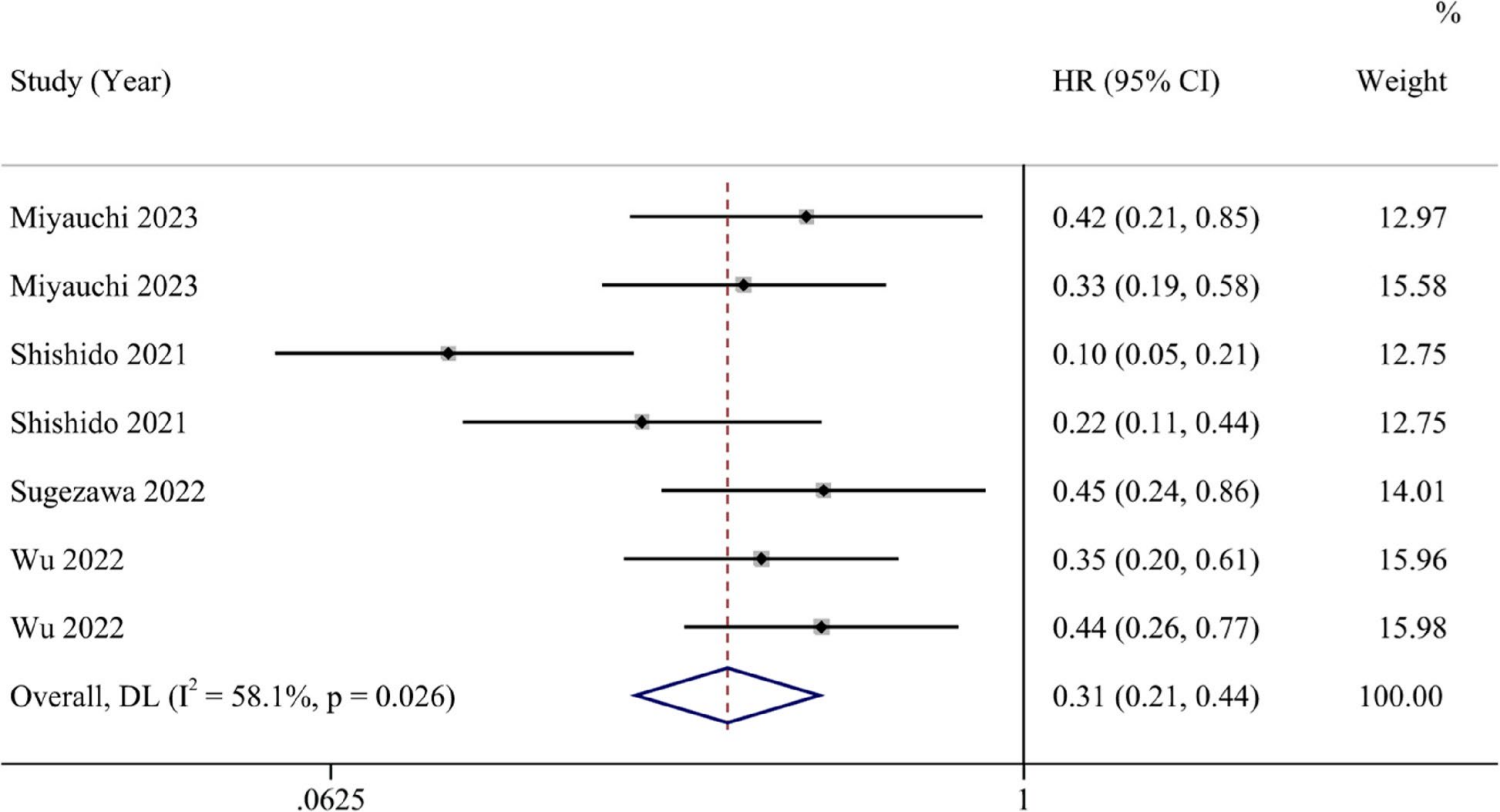
Forest plot of the pooled overall survival between the ferroptosis-promoting group and the ferroptosis-inhibiting group after excluding one study with a large bias

A total of 8 studies reported HRs and 95% confidence intervals for OS. The forest plot indicates that the ferroptosis-promoting group had better OS compared to the ferroptosis-inhibiting group (HR 0.43, 95% CI 0.22–0.83). Data analysis reports substantial heterogeneity (I^2^ = 87.8%, 95% CI 45.6%-94.7%) (Fig. 2). After conducting sensitivity analysis, we found that the study by Song et al. might introduce significant bias. After excluding this study and reanalyzing the data, the results showed that the ferroptosis-promoting group had better OS compared to the ferroptosis-inhibiting group (HR 0.31, 95% CI 0.21–0.44), with decreased heterogeneity (I^2^ = 58.1%, 95% CI 0%-82.7%), indicating moderate heterogeneity (Fig. 3).

**Fig. 4:**
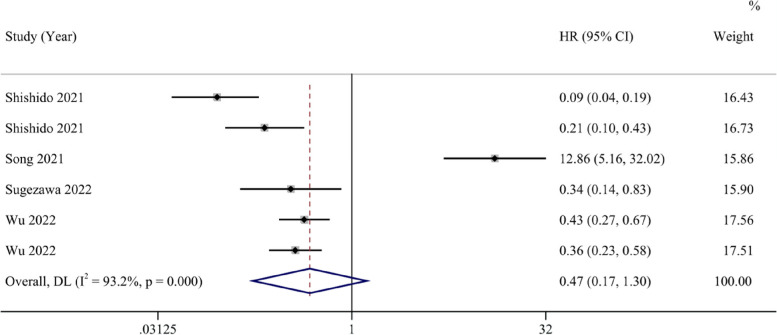
Forest plot of the pooled progression-free survival between the ferroptosis-promoting group and the ferroptosis-inhibiting group

**Fig. 5:**
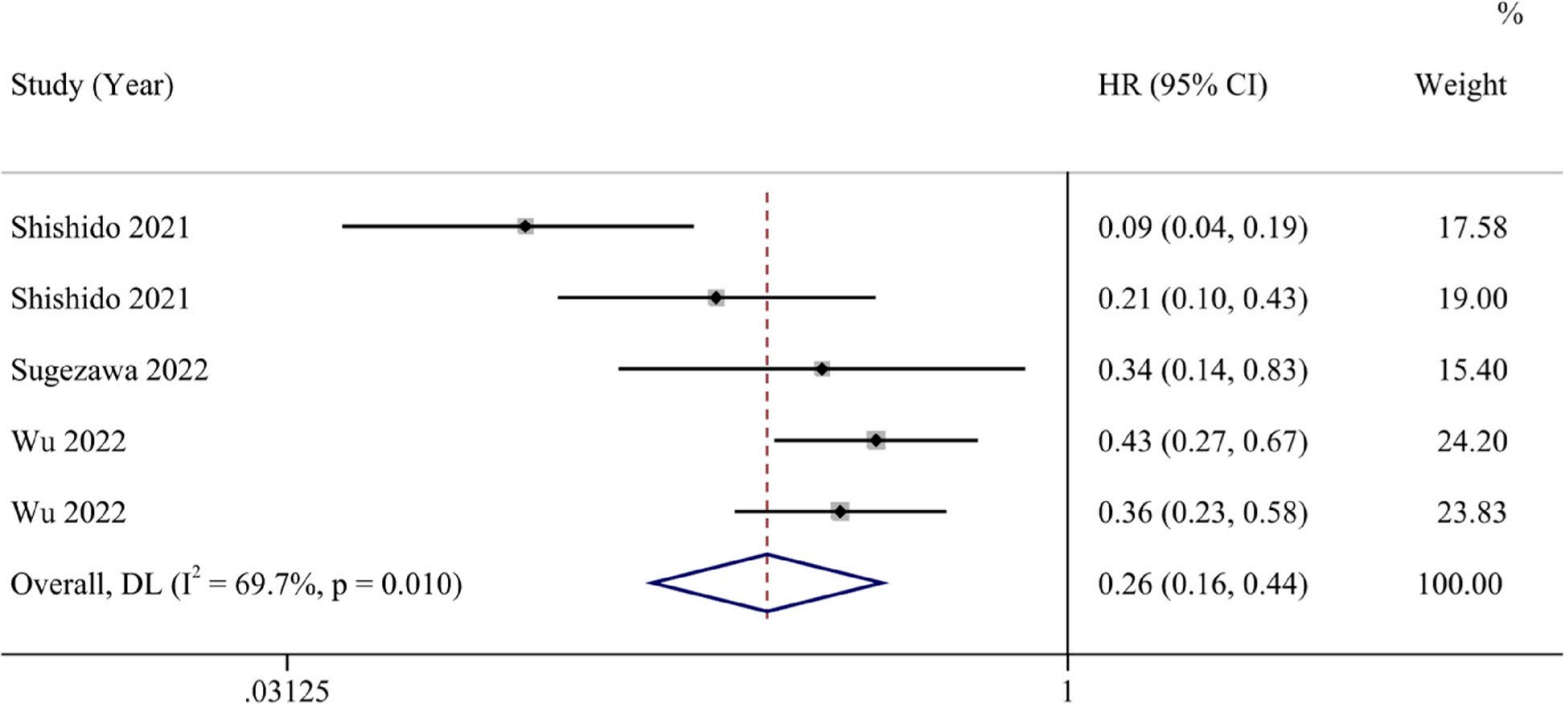
Forest plot of the pooled progression-free survival between the ferroptosis-promoting group and the ferroptosis-inhibiting group after excluding one study with a large bias

Six studies reported HRs and 95% CIs for PFS. The analysis results suggest that the ferroptosis-promoting group had better PFS compared to the ferroptosis-inhibiting group (HR 0.47, 95% CI 0.17–1.30), although it was not statistically significant. Data analysis reports high heterogeneity (I^2^ = 93.2%, 95% CI 43.5%-97.5%) (Fig. 4). After conducting sensitivity analysis, similar to the OS results, we found that the study by Song et al. might introduce significant bias. After excluding this study, the results showed that the ferroptosis-promoting group had significantly better PFS prognosis compared to the ferroptosis-inhibiting group (HR 0.26, 95% CI 0.16–0.44), with moderate heterogeneity (I^2^ = 69.7%, 95% CI 0%-89.6%), and the results were statistically significant (Fig. 5).

Separate meta-regression analyses for OS and PFS results revealed that covariates such as country of study, ferroptosis-related gene, the effect of genes on ferroptosis, and type of cancer had no influence on the results.

### Risk of bias in studies

All included studies underwent a risk of bias assessment following the guidelines recommended by the Cochrane Handbook, which includes five bias domains. We classified 2 studies as having low bias risk (2/8, 25%), indicating low bias risk across all domains. Five studies exhibited some lower risk (5/8, 62.5%), suggesting mild uncertainty in at least one domain but no definite high risk. One study had a high risk (1/8, 12.5%), indicating high bias risk in more than one domain. No studies presented a higher risk overall. The reasons for non-low bias risk were predominantly due to incomplete outcome data (9/14, 64%). In multiple lower risk studies, the reason for uncertain bias in other domains was the lack of reported cut-off values. We believe that different cut-off values can introduce a certain degree of bias into study results, which may affect the interpretation of the results of the study Moreover, we excluded a study of Song, which have introduced a large bias because its results were not reported clearly and correctly with low credibility. We conducted a thorough review of their experimental procedures and relevant sensitivity analysis, concluding that it could affect the overall bias risk of the study. After excluding the study by Song et al., the Egger tests for OS and PFS had *p*-values of 0.20 and 0.205, respectively, indicating no significant publication bias.

## Discussion

To the best of our knowledge, this systematic review represents the pioneering effort to explore the correlation between ferroptosis and cancer prognosis. Through a comprehensive meta-analysis, we aimed to determine whether ferroptosis influences cancer prognosis and its potential applicability as a therapeutic target. The hallmarks of tumorigenesis encompass the evasion of regulatory cell death, unbridled proliferation, and cellular immortality [26, 27]. The resistance exhibited by cancer cells poses a formidable challenge in cancer treatment, as conventional chemotherapy agents often fall short in inducing effective cell death [28]. Ferroptosis emerges as a promising strategy to overcome this resistance [27]. Nevertheless, ferroptosis assumes a dual role in the context of anti-tumor immunity. CD8 + T cells, for instance, can secrete Interferon-γ to promote ferroptosis in cancer cells, while ferroptotic cancer cells can reciprocally enhance the maturation of dendritic cells and macrophage efficiency [13]. However, it’s worth noting that some T helper cell subsets and CD8 + T cells can themselves undergo ferroptosis, thereby tempering the overall impact of ferroptosis on anti-tumor immunity [13].

In our study, we have uncovered that the promotion of ferroptosis in cancer cells serves as a protective factor for cancer patient prognosis. In our analysis of OS, involving eight studies, the results indicate that patients in the group where ferroptosis is promoted exhibit improved overall survival rates compared to the group where it is inhibited (HR 0.43, 95% CI 0.22–0.83). Following a sensitivity analysis, we observed certain biases in the study conducted by Song et al. Upon a thorough review of the research, we discovered that this study found ZFP36 can express in both tumor and para-carcinoma tissues, and the expression of ZFP36 was higher in para-carcinoma tissues Elevated ZFP36 expression inhibits ferroptosis, consequently leading to fewer instances of ferroptosis in the tumor-adjacent tissue, resulting in better patient prognoses. However, in the other studies included, ferroptosis-regulating genes were all found to be overexpressed or suppressed in tumor tissue instead of tumor-adjacent tissue. Meanwhile, the low accuracy of results from the study of Song et al. can introduce bias to our study. So we exclude this particular article to assure the quality of our results. Upon its exclusion, patients in the group where ferroptosis is promoted demonstrated better overall survival rates (HR 0.31, 95% CI 0.21–0.44), with reduced study heterogeneity and a higher *p*-value in the Egger test. For this intriguing study, we look forward to future research that directly investigates the role of ZFP36 in tumor tissue and whether it presents contrasting effects on patient prognosis. In our study on PFS, after sensitivity analysis, forest plots indicated that patients in the group where ferroptosis is promoted exhibit improved overall survival rates compared to the group where it is inhibited (HR 0.26, 95% CI 0.16–0.44). The heterogeneity could have raised from the absence of the cut-off values, different countries, the differences of ferroptosis-related genes, the type of cancers and the effect of genes on ferroptosis. After conducting meta-regression, we did not identify covariates including country, ferroptosis-related genes, type of cancer and the effect of genes influencing the results. Considering that 5 of the 8 studies we included did not report the cut-off value, we could not include this in meta-regression, which can lead to potential heterogeneity.

As the pioneering meta-analysis investigating the impact of ferroptosis on cancer patient prognosis, we are pleased to find that it serves as a protective factor for cancer patient prognoses. Ferroptosis, as a novel biological behavior distinct from apoptosis, holds promise as a potential approach in cancer treatment. Currently, we have identified numerous key genes in the ferroptosis pathways, and if ferroptosis proves to be an effective cancer treatment modality, targeting these genes would hold significant clinical relevance. These potential targets included down-regulation of GPX4, ZFP36, SLC7A11, FSP1 expression and up-regulation of HMOX1 expression. Moving forward, there is a promising potential to translate these interventions targeting specific factors into practical applications in clinical therapy. This holds great promise as an exciting new avenue in the realm of cancer bio-therapy.

### Limitation

Despite our rigorous article selection, feature extraction, and analysis, this study has certain limitations. Firstly, we require more clinical research, whether retrospective or randomized controlled studies, to substantiate the favorable impact of promoting ferroptosis in cancer cells on the prognosis of cancer patients, including both OS and PFS, both of which are pivotal for patients’ quality of life. Secondly, the cut-off value is a critical parameter; regrettably, many of the articles we included did not report this metric, making it challenging to assess the extent of bias in prognosis results due to cut-off value variations. We also hope that future related meta-analyses will delve further into the influence of cut-off values.

## Conclusion

This meta-analysis, by comparing the promotion and inhibition of ferroptosis in cancer patients, reveals that fostering ferroptosis in cancer cells is a protective factor for cancer patient prognosis. Ferroptosis-related genes hold the potential to become novel biomarkers for targeted therapy, and promoting ferroptosis in cancer cells could represent a new and effective approach to cancer treatment.

### Supplementary Information

Supplementary Material 1.

Supplementary Material 2.

## Data Availability

To ensure transparency and reproducibility of the study, all data generated or analyzed during this study are included in this published article and its supplementary information files. The datasets used and analyzed during the current study are available from the corresponding author on reasonable request. Please note that data sharing is intended for academic research purposes only and not for other purposes.
