# Supplementary material for: Ferroptosis is a protective factor for the prognosis of cancer patients: a systematic review and meta-analysis
Source: BMC Cancer. 2024 May 17;24:604. doi: 10.1186/s12885-024-12369-5 (PMC11102205; doi:10.1186/s12885-024-12369-5)
Supplement: Supplementary file 2 — Supplementary Material 2. [file 12885_2024_12369_MOESM2_ESM.docx]

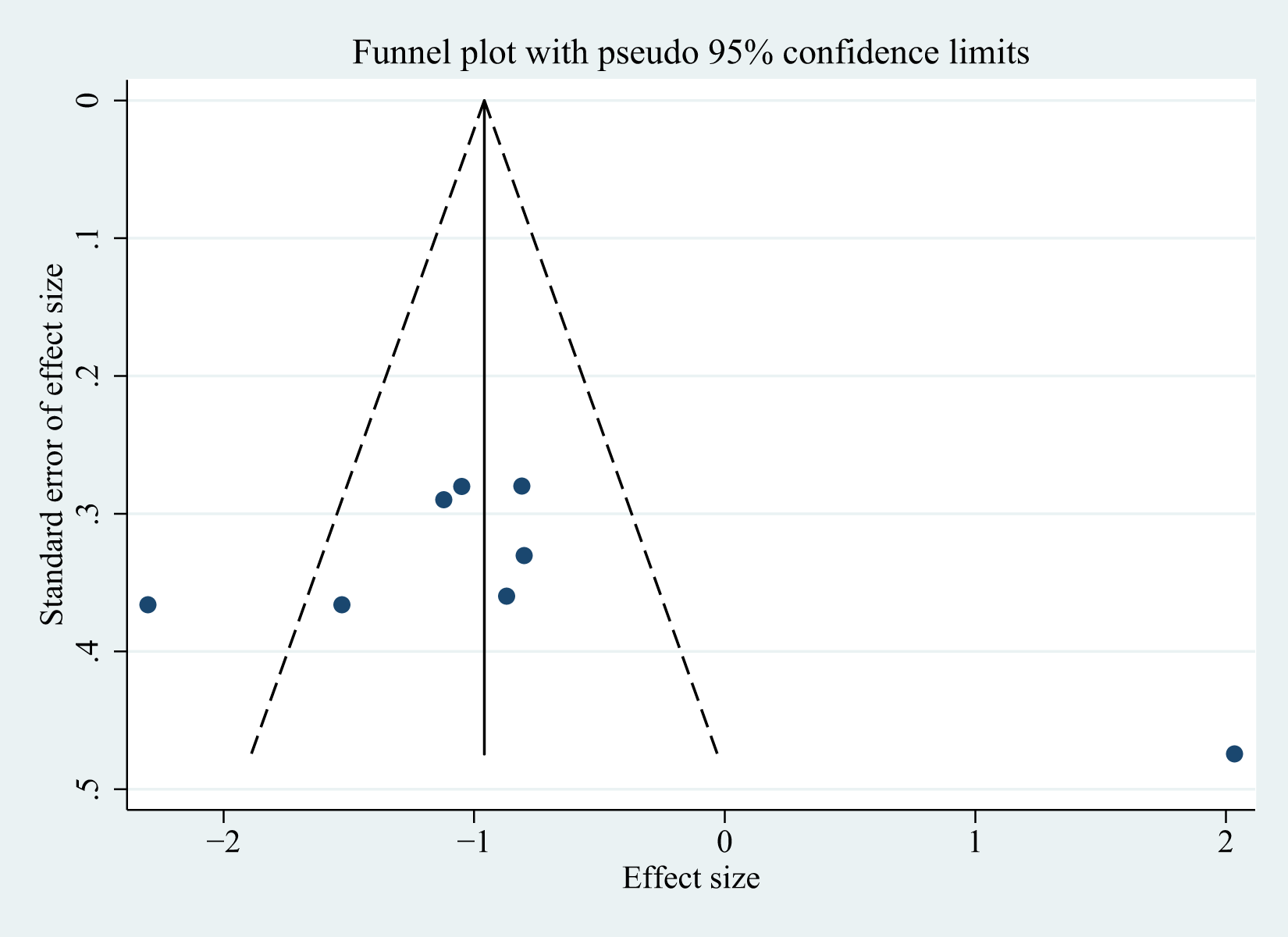


Figure 1: Funnel plot of studies included in OS meta-analysis.


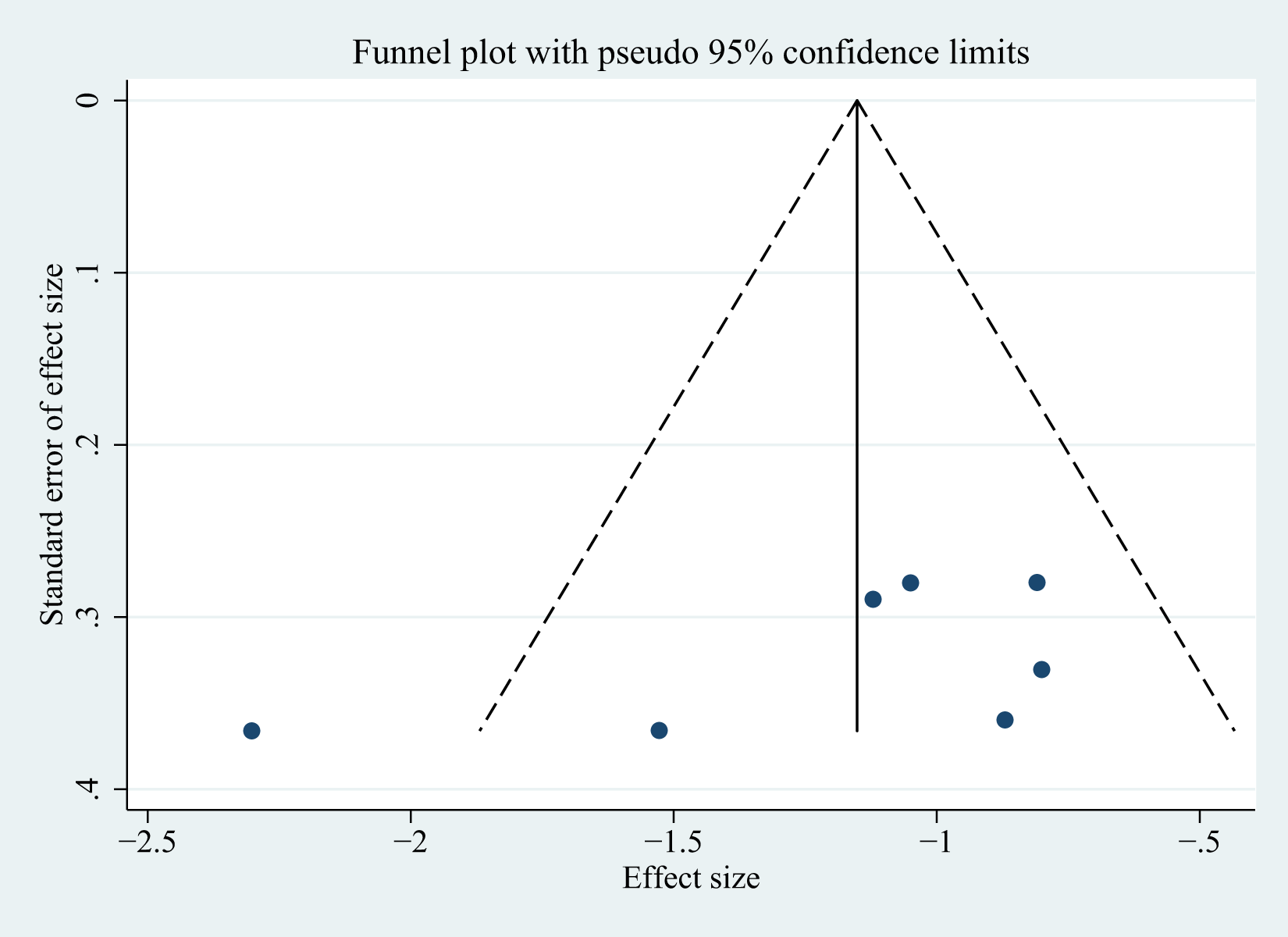


Figure 2: Funnel plot of studies included without the study of Song in OS meta-analysis.


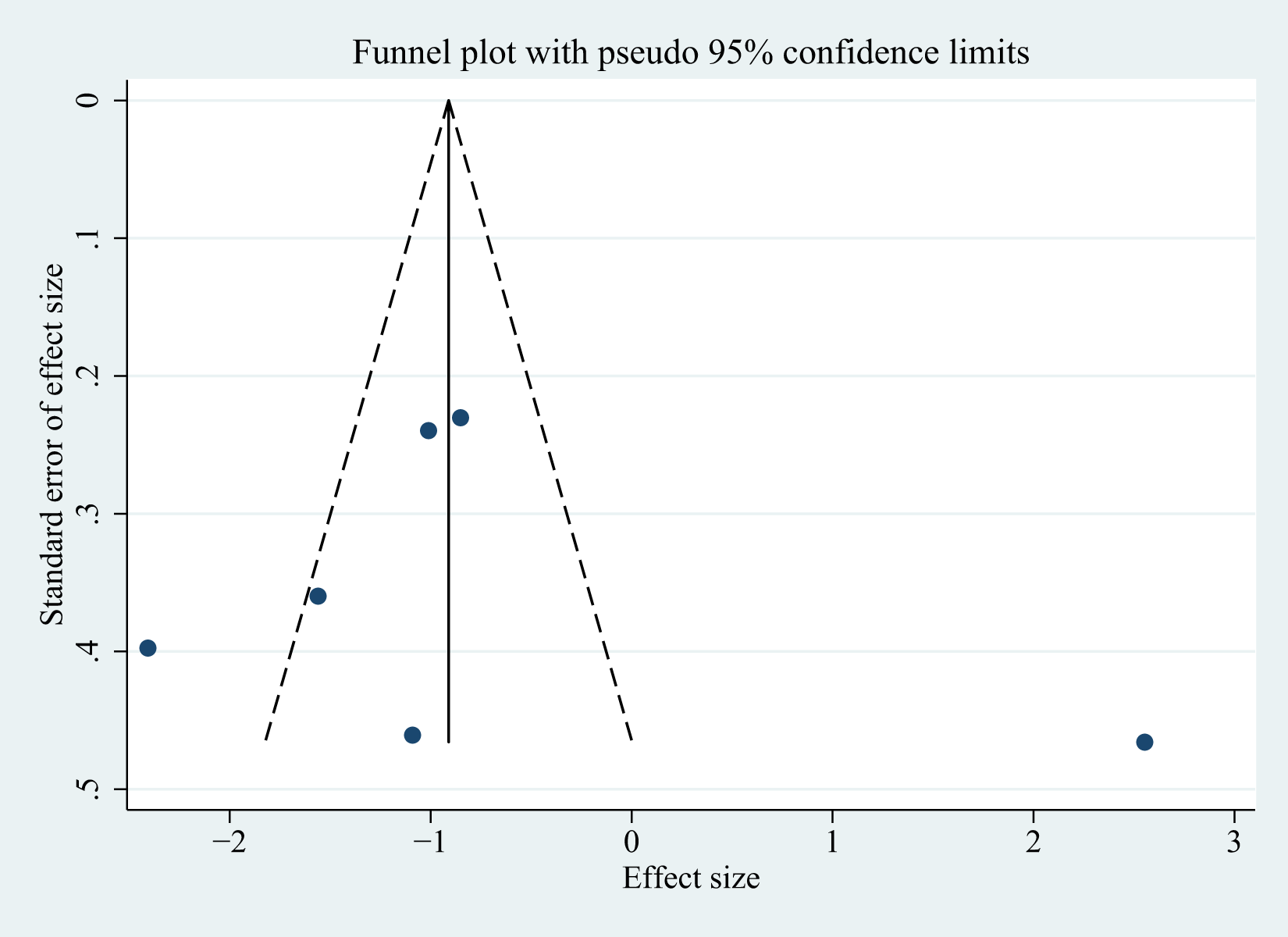


Figure 3: Funnel plot of studies included in PFS meta-analysis.


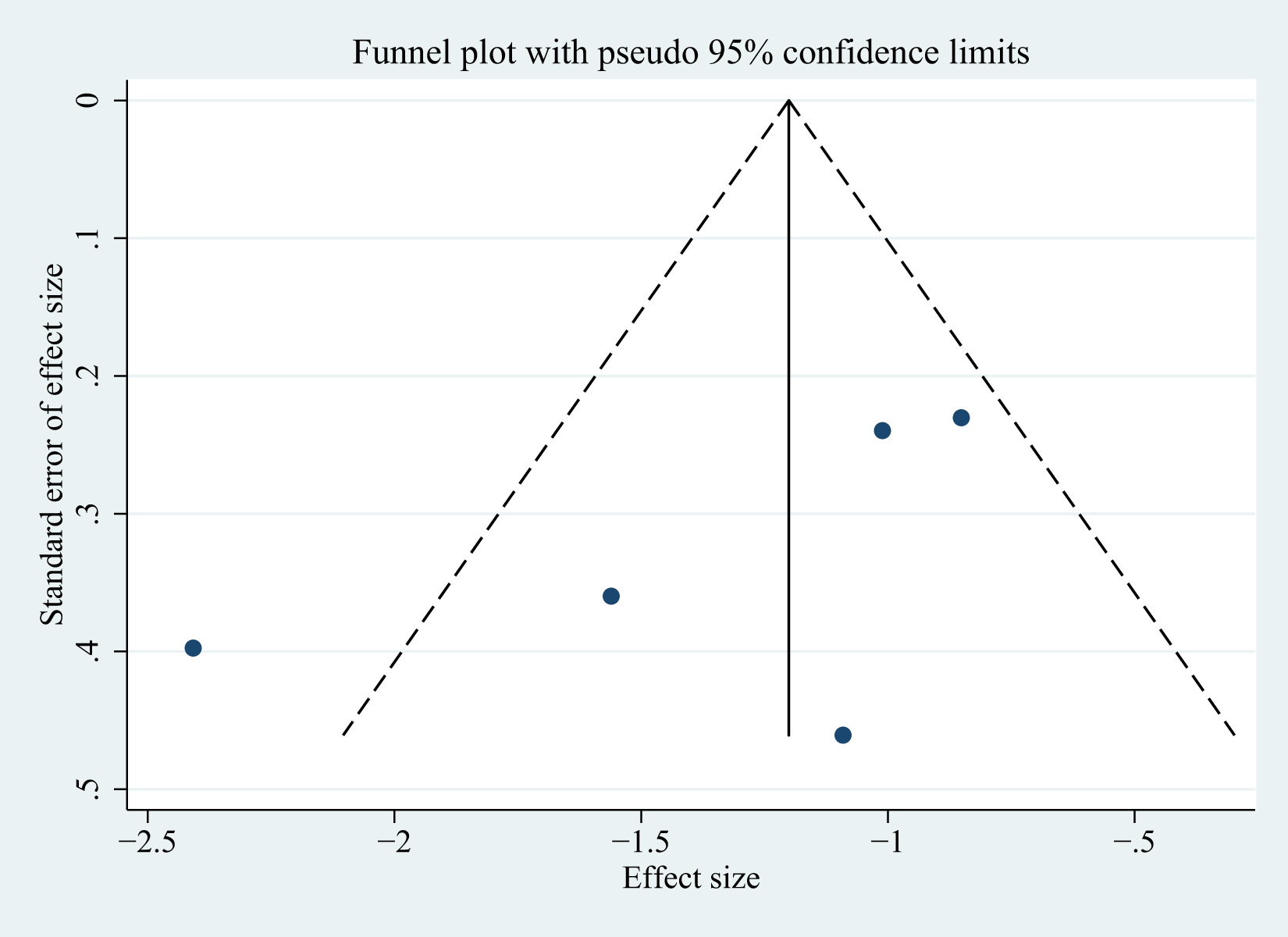
 Figure 4: Funnel plot of studies included without the study of Song in PFS meta-analysis.


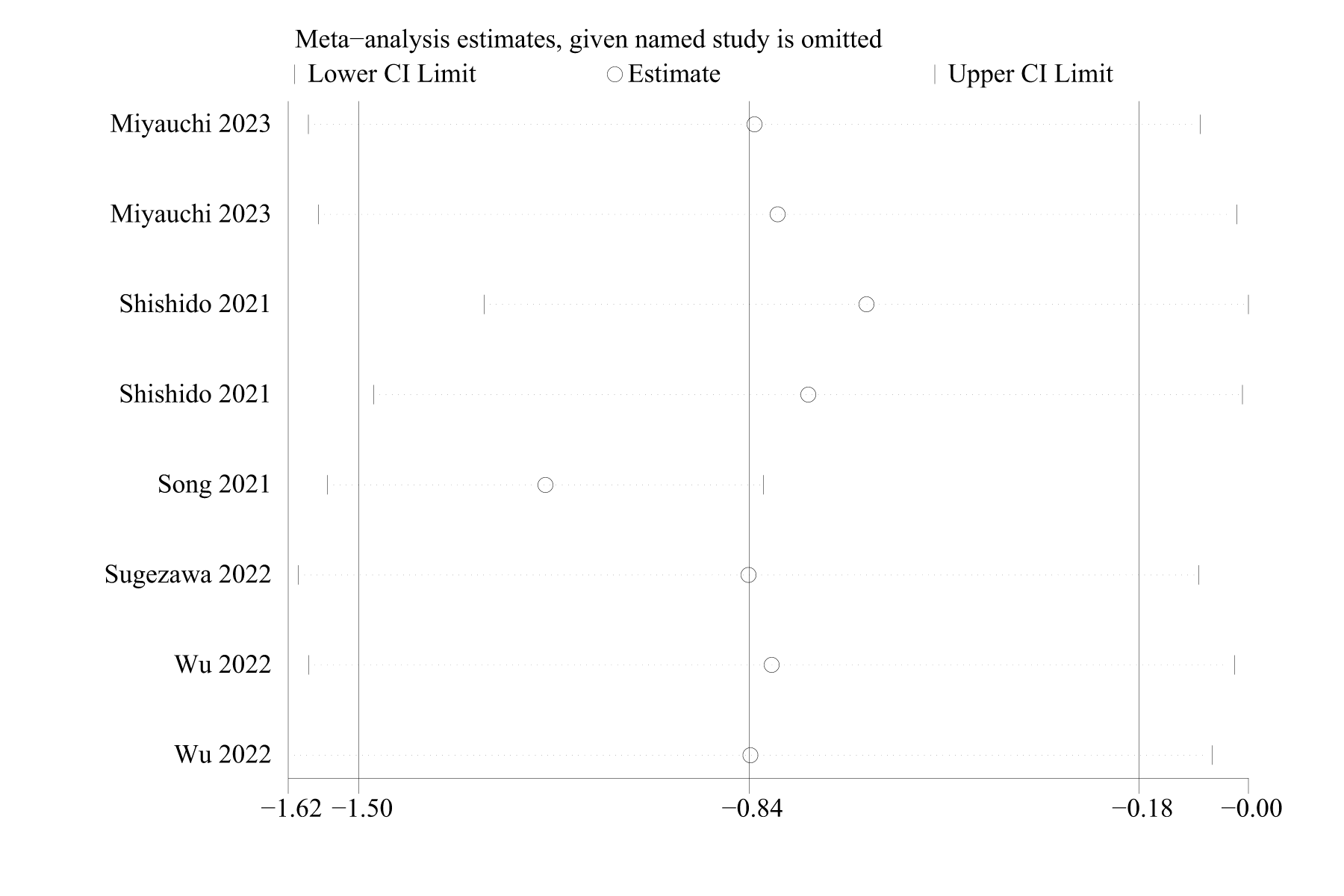


Figure 5: Sensitivity analysis of OS results in meta-analysis.


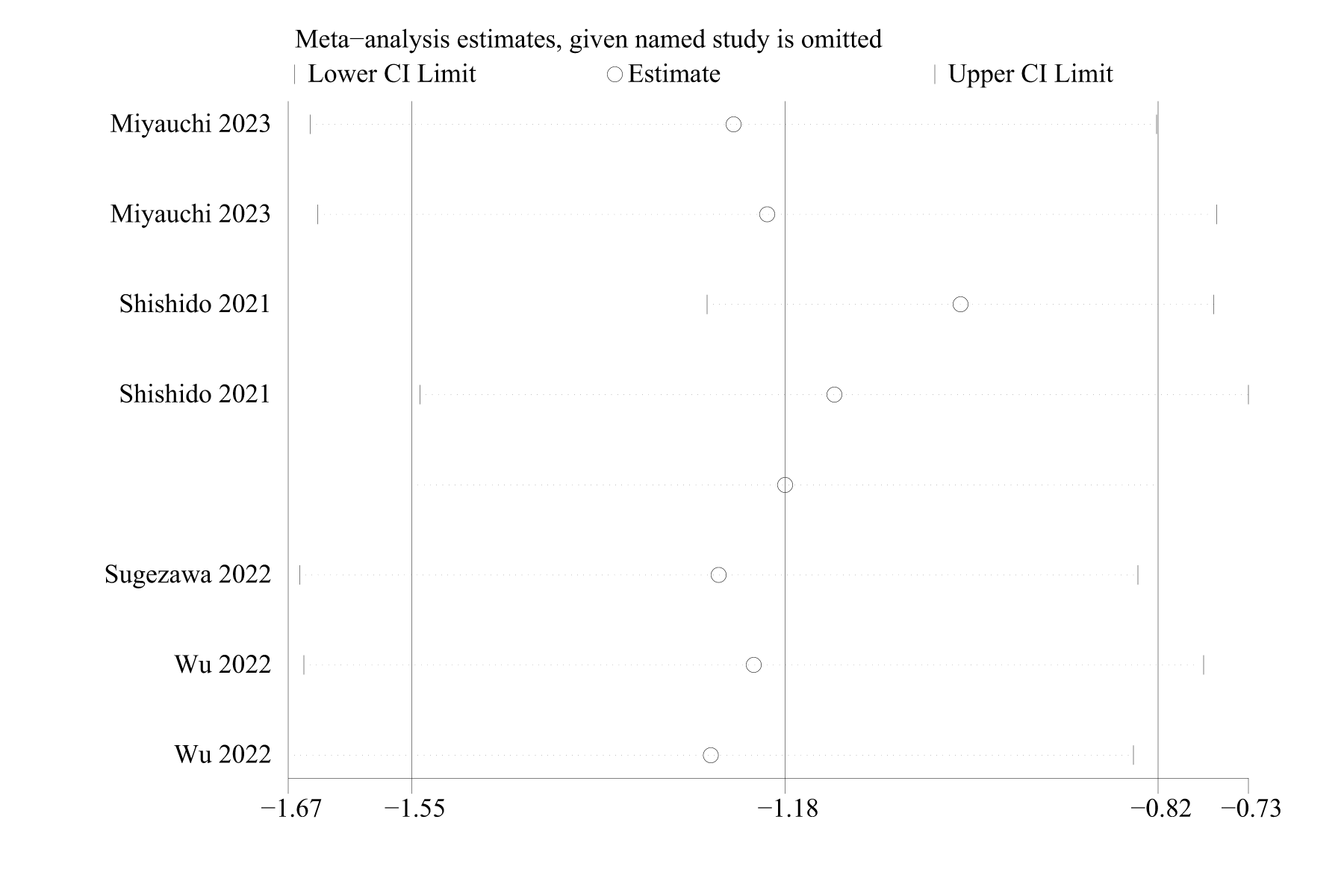


Figure 6: Sensitivity analysis of OS results in meta-analysis without the study of Song.


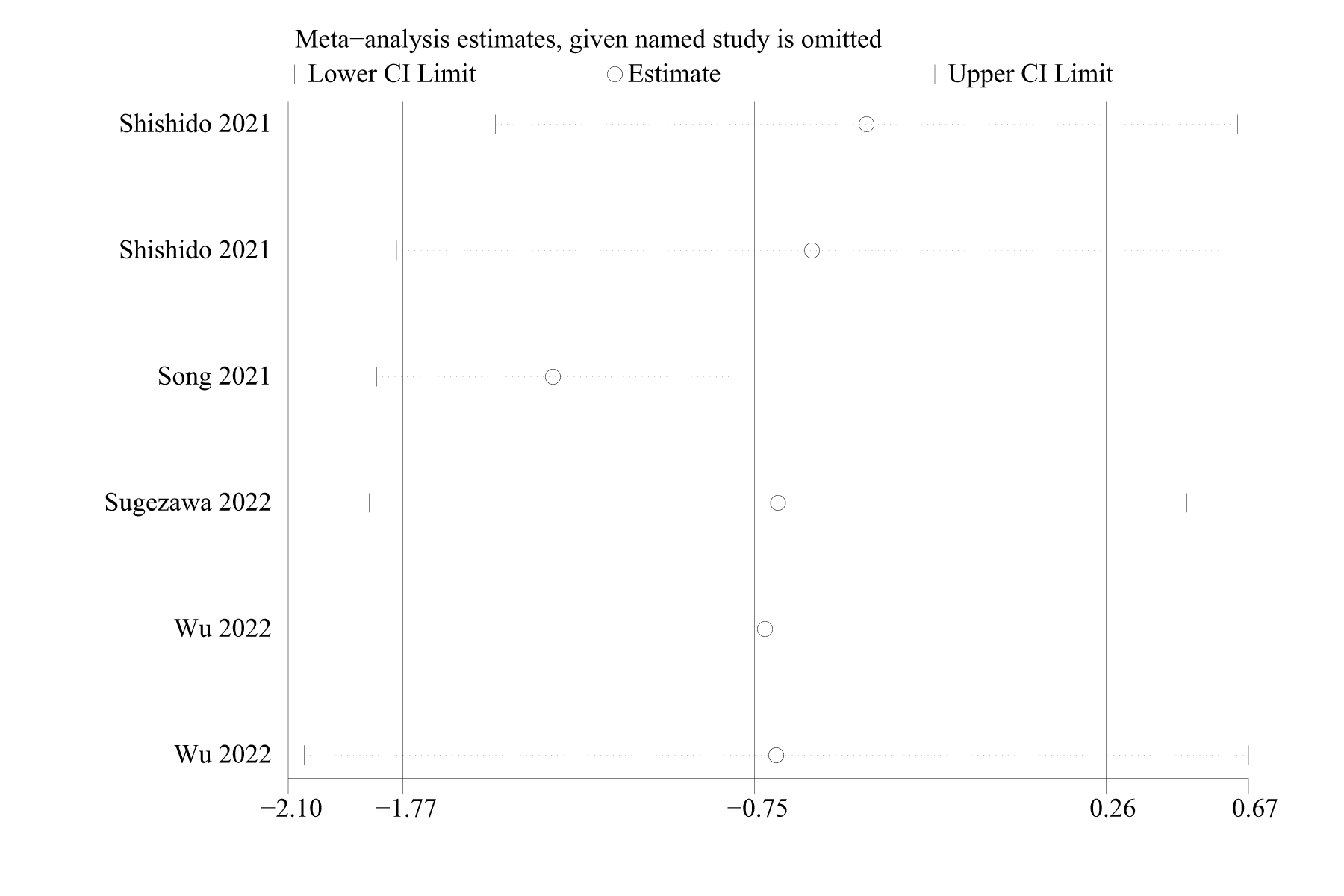


Figure 7: Sensitivity analysis of PFS results in meta-analysis.


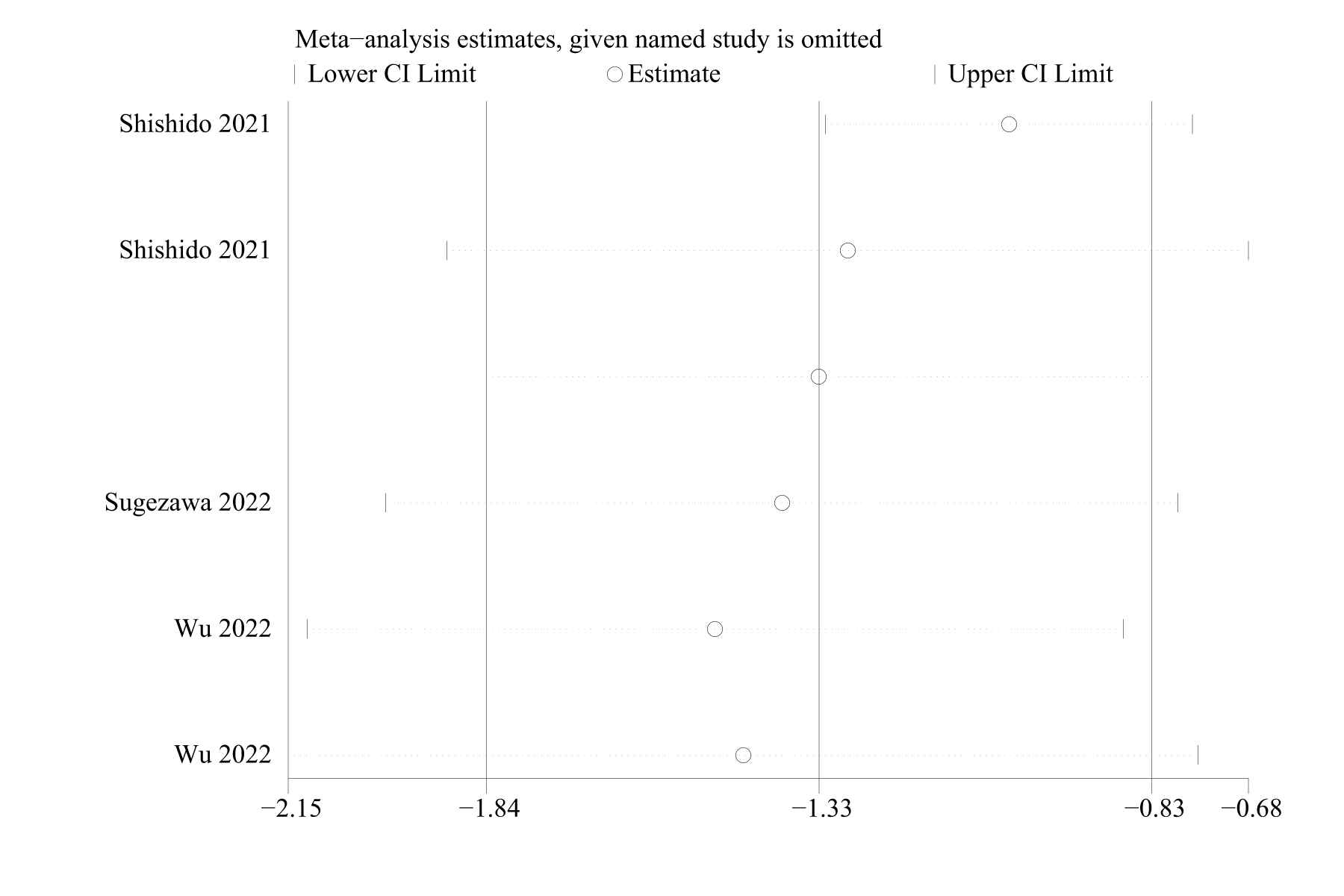


Figure 8: Sensitivity analysis of PFS results in meta-analysis without the study of Song.


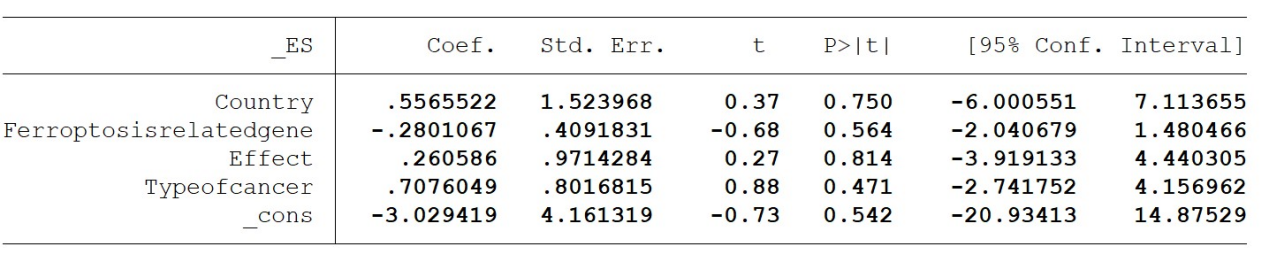


Figure 9: Results of meta-regression of included studies reporting OS outcome.


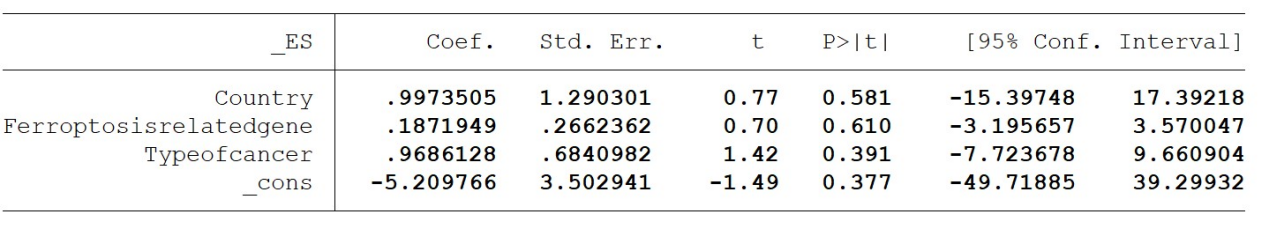
 Figure 10: Results of meta-regression of included studies reporting PFS outcome.


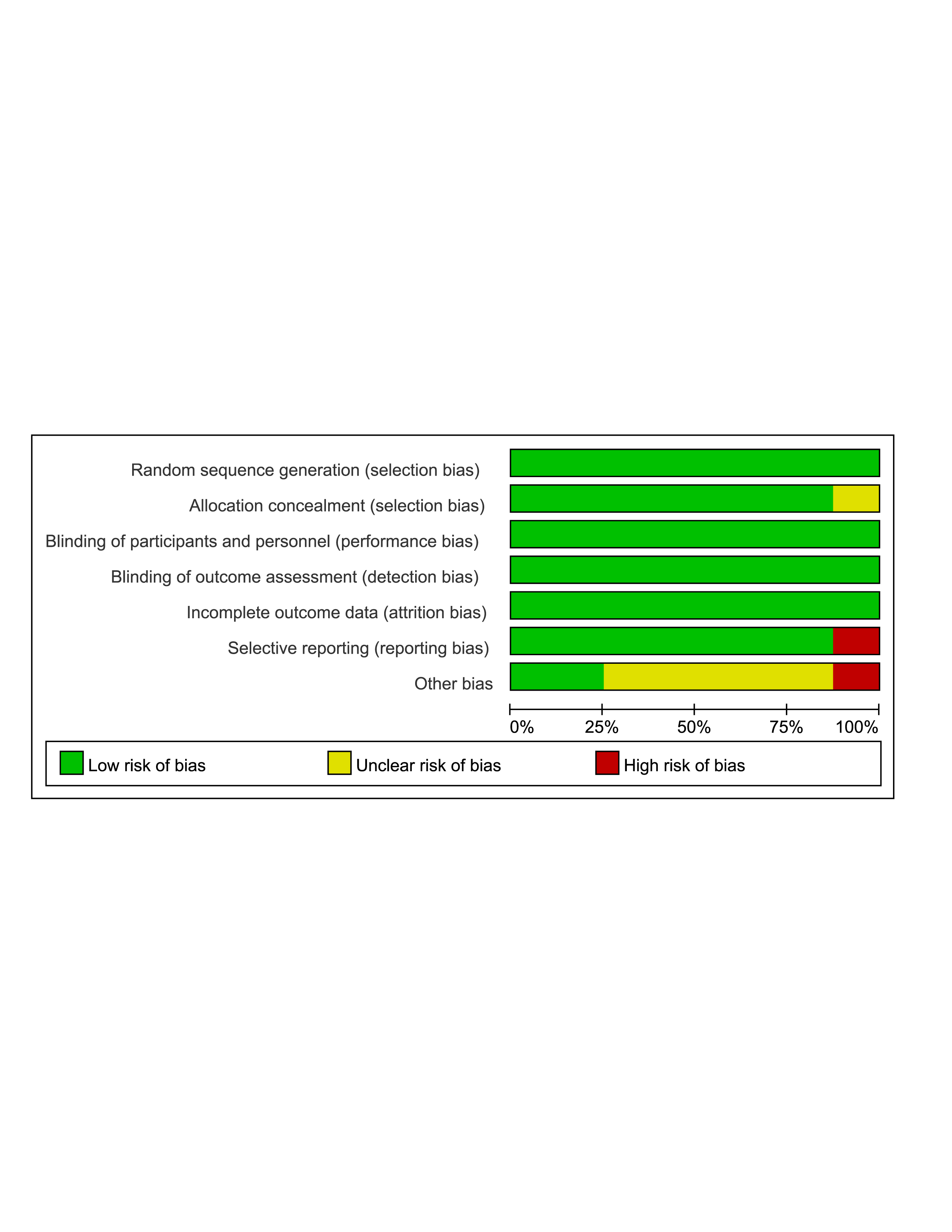


Figure 11: Methodological quality summary and risk of bias assessment statistics.


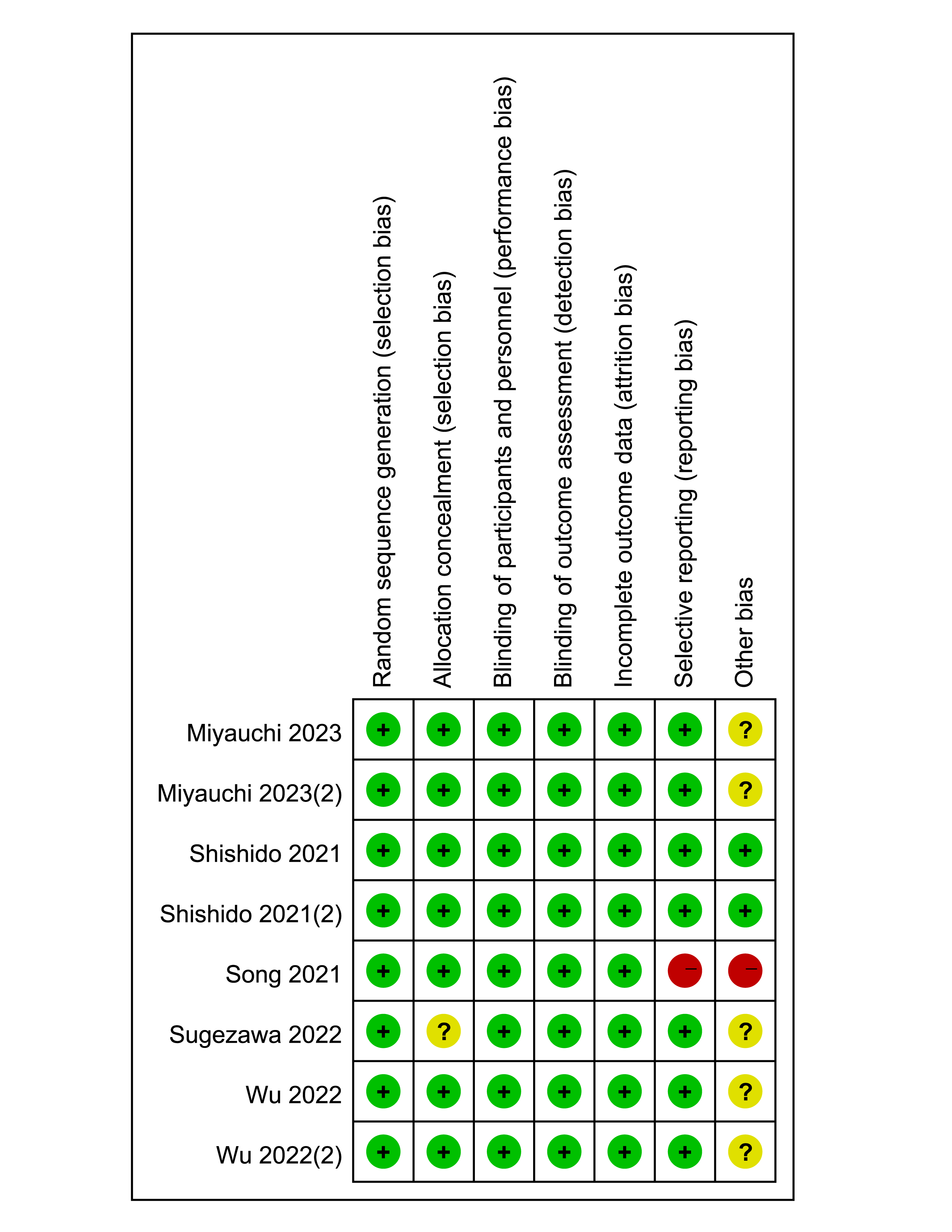


Figure 12: Methodological quality summary and risk of bias assessment.
